# Shorter planning depth and higher response noise during sequential decision-making in old age

**DOI:** 10.1038/s41598-023-33274-0

**Published:** 2023-05-11

**Authors:** Johannes Steffen, Dimitrije Marković, Franka Glöckner, Philipp T. Neukam, Stefan J. Kiebel, Shu-Chen Li, Michael N. Smolka

**Affiliations:** 1 Department of Psychiatry and Psychotherapy, Technische Universität Dresden, Dresden, Germany; 2 Department of Psychology, Technische Universität Dresden, Dresden, Germany; 3 Department of Psychiatry, Icahn School of Medicine at Mount Sinai, New York, NY USA

**Keywords:** Psychology, Human behaviour

## Abstract

Forward planning is crucial to maximize outcome in complex sequential decision-making scenarios. In this cross-sectional study, we were particularly interested in age-related differences of forward planning. We presumed that especially older individuals would show a shorter planning depth to keep the costs of model-based decision-making within limits. To test this hypothesis, we developed a sequential decision-making task to assess forward planning in younger (age < 40 years; n = 25) and older (age > 60 years; n = 27) adults. By using reinforcement learning modelling, we inferred planning depths from participants' choices. Our results showed significantly shorter planning depths and higher response noise for older adults. Age differences in planning depth were only partially explained by well-known cognitive covariates such as working memory and processing speed. Consistent with previous findings, this indicates age-related shifts away from model-based behaviour in older adults. In addition to a shorter planning depth, our findings suggest that older adults also apply a variety of heuristical low-cost strategies.

## Introduction

Making sequential decisions to pursue long-term goals is an implicit routine task for humans of all ages. One option to approach such complex decision-making scenarios is to use model-based forward planning^1,2^. By ‘model-based’, we refer to decision-making relying on planning based on a representation of the individuals’ environment, i.e. an internal model of available actions, possible states, probabilities of transitioning between states as well as outcome probabilities of entering a state. One obvious challenge of model-based forward planning is the question of how far one should plan ahead in order to reach one's goal. If the goal is temporally distant, it is crucial to consider the long-term consequences of the available actions: an action might make good short-term progress towards the goal but lead to adverse outcomes in the long run^3^. One option is to exhaustively plan through all possible action sequences and compare their overall reward with each other. However, this strategy would span a decision tree with an exponentially increasing number of action sequences for increasing planning depth. This would soon become infeasible, especially because in real world scenarios, the action-outcome relationship usually is not deterministic but probabilistic and one action may have several possible outcomes which increases the amount of possible action sequences further. To deal with these complexities in light of limited cognitive resources, especially in old age^4,5^, people utilize different strategies to reduce the length of action sequences that have to evaluated, i.e. to prune the decision tree. Therefore, the choice of planning depth is naturally linked to the basic trade-off between predictive accuracy and computational complexity. The planning gets more complex and effortful with every step of deeper planning. However, at the same time, with deeper planning depth, people are more likely to find a behavioural policy enabling them to reach their goal. In our study, we focused on the question, how planning depth is modulated by older age.

Although we previously have observed effects of aging on sequential decision making with older adults performing particularly worse when crucial outcomes only occurred in several decision states later^3^, it is unclear whether older adults would opt for a shorter planning depth in situations when individuals can plan their own decision steps in a probabilistic environment with a fully transparent task structure. So far, no study has investigated the effects of aging on forward planning depth in sequential decision-making. However, evidence from two related research branches reveals impairments in model-based decision-making in old age. Firstly, classical sequential problem-solving tasks like the Tower of London Task^6^ or the Tower of Hanoi Task^7^ require participants to plan ahead to find an action sequence to the target configuration with as few actions as possible. The common finding is that older adults require more actions to reach the given target configuration^8^. This suggests that older adults might not have planned far ahead enough to find the shorter sequence. However, these tasks do not directly assess planning depth. Moreover, they address a specific problem-solving scenario with a deterministic environment and a single given target state, which limits external validity. Secondly, the relative influence of model-based and model-free control on human decision-making has been investigated in reward-based sequential decision-making tasks like the two-stage Markov task^9^. While these studies found a decreased contribution of model-based control to choices of older adults^10–12^, they also do not differentiate computational parameter values of model-based control like planning depth. Nevertheless, both strands of evidence suggest that older compared to younger adults demonstrate reduced forward planning capabilities.

Additionally, forward planning involves several fluid cognitive abilities like working memory, processing speed and executive control^13^. These have been shown to influence model-based learning^11,14–16^ and are classical examples for significant cognitive decline in old age starting from early adulthood^5^. In related neuropsychological tasks, older adults usually demonstrated both, an overall slowing as well as less accurate responses^17^. Previous findings of lower performance in model-based control in old age might be confounded with these general cognitive abilities. Similarly, they might also explain differences in planning depth. We therefore included indicators of working memory and processing speed in assessments of forward planning as potential covariates.

In order to assess potential age-related differences in planning depth between younger and older adults, we designed a sequential decision-making task, the Space Adventure Task (SAT), which required participants to use model-based planning in order to make beneficial choices. The task further allowed to differentiate between different planning depths based on participants’ choices. Choices in the SAT were modelled with a reinforcement learning (RL) agent model which in turn allowed us to infer planning depths with hierarchical Bayesian inference of free model parameters (see Methods section for details). To motivate participants, deeper planning yielded higher rewards. Moreover, we varied the degree of randomness in state transitions, i.e. the predictability of outcomes, to explore how participants adapt planning when facing different levels of uncertainty. To investigate to what extent differences in planning depth could be explained by classical constructs of cognitive performance, participants underwent a neuropsychological assessment for processing speed and spatial working memory.

Based on previous research, we hypothesized that in the SAT, older compared to younger adults should demonstrate reduced forward planning capabilities indicated by lower scores and a lower inferred planning depth. Regarding outcome certainty, we presumed that a less predictable environment should lead to a reduction in planning depth. Finally, we expected performance measures for the two assessed general cognitive abilities to be positively associated with planning depth while not fully explaining group differences in planning depth.

## Methods

### Participants

Twenty-seven older adults (13 women, age above 60 years: M = 68.8) and twenty-five younger adults (7 women, age below 40 years: M = 26.4) took part in the experiment. The study was conducted as an associated experiment for the larger research project "Aging and neuromodulation of forward planning under uncertainty" in the collaborative research centre funded by the German Research Foundation (DFG SFB 940). Prior to the appointment, participants were telephone screened for potential exclusion due to psychiatric or neurological illness. The assessment consisted of a sociodemographic questionnaire, an eyesight test, a neuropsychological test battery and the SAT. The overall procedure took around 2‒2.5 h and participants were compensated with 20 Euros plus a maximum of additional 10 Euros depending on their performance in the SAT. Ethic approval in accordance with the Helsinki declaration was granted by the ethic committee of the TU Dresden, Germany (EK 514122018). All participants signed informed consents before the start of study participation. Demographic characteristics and basic cognitive covariates are depicted in Table 1. Age ranged from 17 to 38 years for younger adults and from 61 to 75 years for older adults. Groups did not differ significantly in gender distribution according to Pearson’s chi-squared test, *χ*^2^(1) = 2.23, *p* = .136.

### Cognitive tests

Cognitive covariates were assessed with three computerized tasks. The Spot-a-Word Test (SAW) examined verbal knowledge as an indicator for crystalline intelligence and the Identical Pictures Task (IDP) measured perceptual processing speed as an indicator for fluid intelligence^18^. Thirdly, the first subtask (location memory condition across both load levels) of the Spatial Working Memory Task (SWM) was used to test for spatial abilities^19^. For brief descriptions of the tasks, see Figs. S1–S3. In each test, reaction time (RT) and number of correct responses were measured. Trials with reaction time below 150 ms were excluded. Performance was calculated as achieved percentage of maximum possible number of correct responses. In line with previous evidence^5^, independent *t*-tests of group means revealed that older adults showed a significantly lower performance in the tasks associated with fluid intelligence, the SWM (*t*(32.06) = 3.70, *p* < .001) and the IDP (*t*(50) = 7.62, *p* < .001). Similarly, as expected, older adults showed superior results for crystalline intelligence in the SAW, as indicated by a Mann–Whitney-U test (used because of non-normal distributions; *Z* = − 2.47, *p* = .013).

### Space adventure task

**Figure 1:**
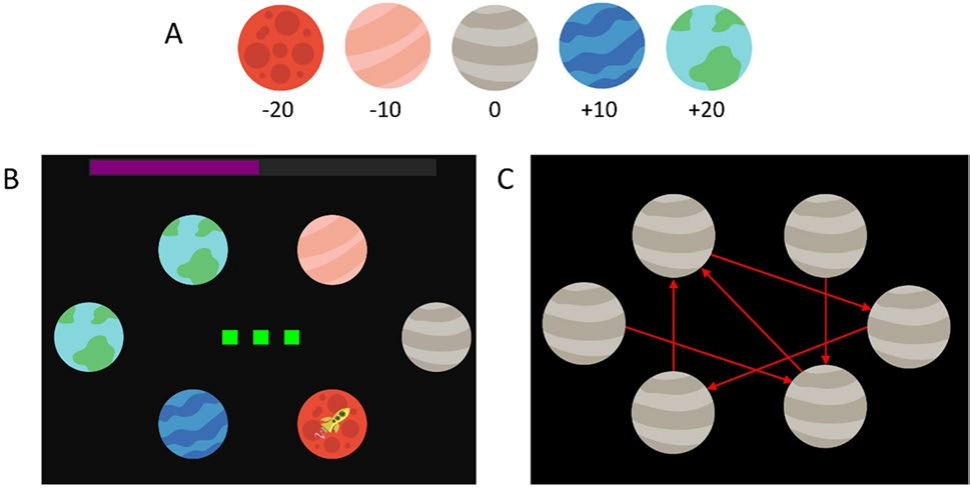
Schematic of the Space Adventure Task. (**A**) Five planet types with their respective reward values for visiting a planet of that type. (**B**) Example mini-block with three steps (green squares) and low noise (black background). There are six planets with the yellow rocket symbol indicating the current location. The starting position and planet configuration varied across mini-blocks. The fuel bar at the top of the screen showed the remaining amount of fuel. Importantly, the fuel level was carried over between mini-blocks. (**C**) Travel pattern of the jump action. This pattern was once presented to participants for memorization and then practiced during training.

The SAT is a sequential decision-making task based on a task first described by Huys et al.^20^. Participants navigated a spaceship through various planetary systems. Each system was a configuration of six planets, each being one out of five possible planet types. Participants had to spend fuel to travel between planets but could gain fuel by arriving at specific planet types (Fig. 1A). The task goal was to accumulate as much fuel as possible throughout the experiment consisting of the same sequence of *N* = 100 mini-blocks for every participant.

In each mini-block, participants were presented with a specific planet configuration and starting position of their spaceship. A bar at the top indicated the current amount of fuel accumulated so far, as well as the number of remaining actions that can be performed during the mini-block (Fig. 1B). The mini-blocks were designed in a way that participants had to use forward planning to find the route leading to the maximum possible fuel gain within a specific number of actions (two or three steps).

At each step, participants could choose to either (i) move to the next planet in a clockwise fashion, or to (ii) jump to a specific non-neighbouring planet. Travelling to the next planet into clockwise direction cost two units and jumping cost five units of fuel. The target planets for jumping were determined by a given travel pattern (Fig. 1C). Moreover, while moving clockwise was a deterministic action, jumping was uncertain, i.e. jumping was successful only with a specific probability. In case of jump failure, the jump led to one of the two neighbouring planets of the target planet, each with equal probability.

In addition to the available numbers of actions, the jump (transition) uncertainty was varied among mini-blocks. In the low noise condition, jumping succeeded with a 90% probability, while in high noise mini-blocks (indicated by asteroids in the background) it succeeded with a 50% probability. The experiment had a 2-by-2 factorial design with the factors 'steps' (two or three total actions) and 'noise' (low or high uncertainty). The resulting four conditions were assigned to four phases with 25 mini-blocks each. The order of these phases was counterbalanced between participants (see Fig. S4). For each mini-block, choice and RT data was acquired as well as the amount of accumulated fuel as points.

The SAT was implemented in MathWorks MATLAB R2017a and run on a standard PC. Participants controlled the experiment with a computer keyboard. The move action was selected with the right arrow key and the jump action with the 'S' key. Prior to the experiment, participants underwent an extensive training: they were informed about the goal and conditions of the task and were instructed to carefully look for the optimal route of actions to choose in each mini-block. Moreover, they were informed about the effect of jumping uncertainty, but they were not given explicit probabilities but learned success/failure probabilities over time (see also next section). Participants were also tested how well they had memorized the travel pattern for jumps with feedback. Finally, they familiarized themselves with the task procedure during 20 training mini-blocks, 5 mini-blocks per condition. More details on the training procedure can be found in the supplementary material.

### Computational model

We modelled participants' action choices with a mixture model of three single model-based RL agent models with planning depth of 1, 2 and 3 respectively. Each agent had an optimal model of the environment, i.e. it was completely informed about the rules of the task. This environment model entailed the set of available actions $A = \left\{ {^{\prime}move^{\prime},\;^{\prime}jump^{\prime}} \right\}$ and states $S$ (the planet configuration of the mini-block), the transition probabilities $p\left({s}_{t+1}|{s}_{t},{a}_{t}\right)$ for reaching a subsequent state ${s}_{t+1}$ from a given state ${s}_{t}$ with action ${a}_{t}$ as well as the immediate reward of reaching each state $r\left(\mathrm{s}\right)$ indicating the planet types of the current configuration. Here, *t* denotes the trial within a mini-block. To choose the optimal action for a specific state in a specific mini-block, the agents computed the expected cumulative reward for executing each action with an optimal forward planning algorithm (value iteration algorithm; a detailed formulation can be found in the supplementary material) only limited by their planning depth.

Value iteration outputs the expected cumulative reward for executing each action $a$ in the current state $s$ with planning horizon $d$, which is called the state-action- or $Q$-values. These *Q*-values were the essential values for action selection. The higher the relative value of an action was, the higher should have been the probability of selecting that action. Action selection was therefore modelled probabilistically with a softmax function, one of the most universal assumption in the model-based reinforcement learning literature^21^. For our case of two available actions, this corresponded to a sigmoid transformation $\sigma \left(x\right)$ of the difference between the *Q*-values, $\Delta Q\left({s}_{t},d\right)$. Choice probabilities were thus defined as:

1 $$p\left({a}_{t}=\mathrm{^{\prime}}jump\mathrm{^{\prime}}| {s}_{t},d\right)=\sigma \left(\beta *\Delta Q\left({s}_{t},d\right)+\theta \right),$$

2 $${\text{where}}\quad \sigma \left( x \right) = \frac{1}{{1 + e^{ - x} }}$$

3 $$\Delta Q\left( {s_{t} ,d} \right) = Q\left( {a_{t} = ^{\prime}jump^{\prime},s_{t} ,d} \right) - Q\left( {a_{t} = ^{\prime}move^{\prime},s_{t} ,d} \right).$$

Here, the inverse temperature beta ($\beta \in [0,+\infty ]$) controlled the extent to which differences in $Q$-values affected action selection. Higher values of beta represented higher probability to select the action with the highest $Q$-value. If beta $=0$, actions are selected with a constant probability independent of outcomes which helped identify individuals which might have ignored the experimental instructions. The parameter theta ($\theta \in [- \infty ,+\infty ]$) denoted an a priori response bias, where negative values implied a bias towards choosing the deterministic 'move' action, which was incorporated to capture a potential risk-averse tendency of individuals^22^.

Since the state transition probabilities for the jump action $p\left({s}_{t+1}| {s}_{t},{a}_{t}=\mathrm{^{\prime}}jump\mathrm{^{\prime}}\right)$ were not given explicitly during training, we assumed an experience-based learning process of the corresponding state transition probabilities for the high and low noise condition respectively. The belief about the probability that a jump at trial *t* will be successful ${\rho }_{t}=p\left({s}_{t+1}=\mathrm{^{\prime}}target\mathrm{^{\prime}}|{s}_{t},{a}_{t}=\mathrm{^{\prime}}jump\mathrm{^{\prime}}\right)$ was updated using the temporal difference rule:

4 $${\rho }_{t+1}={\rho }_{t}+\alpha \left({o}_{t}-{\rho }_{t}\right),$$

depending on the experienced success $\left({o}_{t}=1\right)$ or failure $\left({o}_{t}=0\right)$ of a jump. The learning rate parameter alpha ($\alpha \in [\mathrm{0,1}]$) modelled how fast participants changed their beliefs about the probability of transition success. Larger values of alpha could also be interpreted as faster forgetting of prior experience and stronger reliance on recent outcomes.

### Planning depth and parameter inference

**Figure 2:**
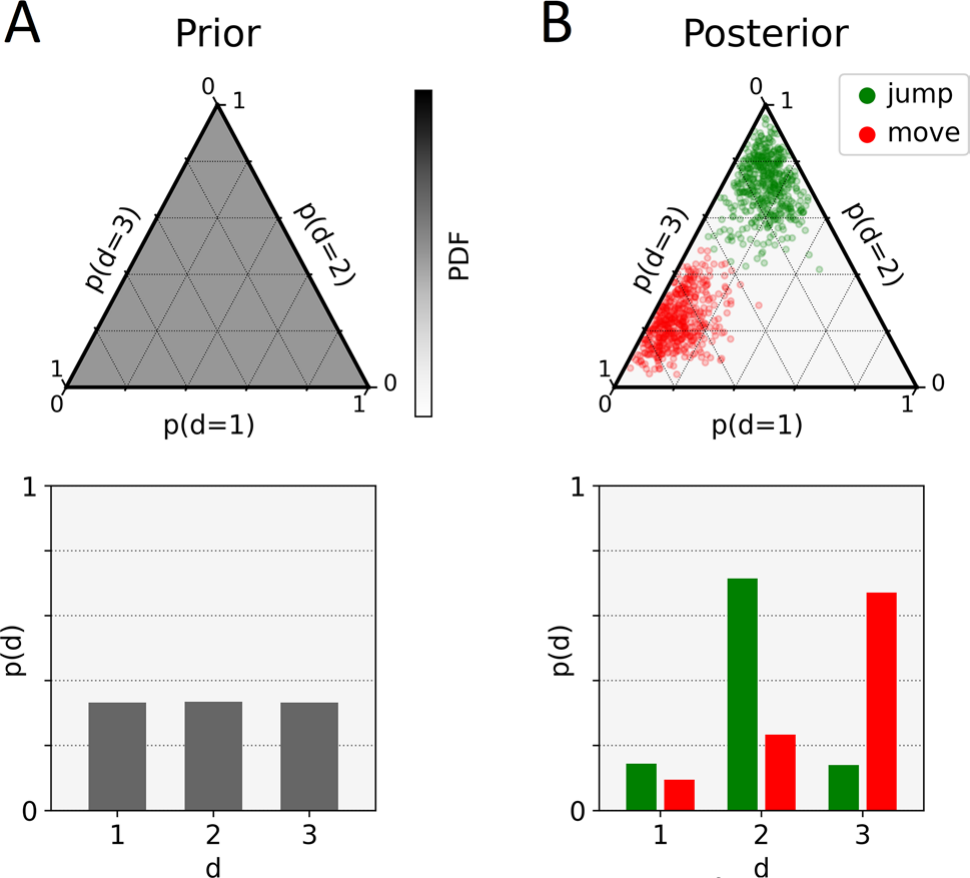
Ternary Plot of Prior Distribution and Marginal Posterior Samples for Planning Depth of one Mini-block*.* (**A**) Uniform Dirichlet prior distribution for planning depth (top) with mean prior corresponding to a uniform categorical distribution (bottom). (**B**) Exemplary marginal posterior samples for planning depth of one mini-block depending on first action being either jump or move (top) where the mean marginal posterior corresponds to a categorical distribution (bottom).

To infer the four described free model parameters, inverse temperature beta, response bias theta, learning rate alpha and planning depth ($d$), from participants' choices, we used a hierarchical probabilistic model of free parameters and the approximate Bayesian inference scheme for computational feasibility. For a detailed description of the parameter inference, please refer to the supplementary material (Eq. S4 ff.). In simplified terms, we combined the above-described choice probabilities of the three RL agent components with planning depth $d\in \left\{\mathrm{1,2},3\right\}$ in the form of a mixture model. The likelihood of our probabilistic model, i.e. the probability of choosing a specific action in a given mini-block, was thus defined as:

5 $$p\left({a}_{b}|{s}_{b}\right)={\sum }_{d=1}^{3}p\left({d}_{b}=d\right)p\left({a}_{b}|{s}_{b},{d}_{b}=d\right)$$

where $p\left({d}_{b}=d\right)$ denotes the probability each planning depth has and acts as a weight of the choice probability of the corresponding agent in the mixture model, where $p\left({d}_{b}=3\right)$ was set to zero for two-stage mini-blocks. Moreover, $p\left({a}_{b}|{s}_{b}\right)$ and $p\left({a}_{b}|{s}_{b},{d}_{b}=d\right)$ are also functions of the model parameters $(\beta ,\theta ,\alpha )$ described in the previous section (compare Eq. S6). These had to be marginalized out in order to retrieve a marginal posterior distribution over planning depth. As illustrated in Fig. 2, the mean of marginal posterior samples of planning depth for one mini-block was a categorical distribution. We calculated the mean of this distribution to get a mean planning depth per mini-block. Importantly, we assumed that forward planning should mostly happen before the first action of each mini-block. Hence, for the model inversion (parameter inference), we have constrained behavioural data only to the first choice in each mini-block being either move or jump.

To account for the limited amount of behavioral data, and for expected within group similarities in participants responses, we designed the probabilistic generative model in a hierarchical fashion with group-level, subject-level and condition-level priors. The parameters beta, theta and alpha were then modelled on the subject-level, while planning depth $d$ was modelled on the mini-block-level. As an analytical solution for the posteriors of the parameters was intractable and also Markov chain Monte Carlo methods were computationally infeasible, we instead used the stochastic variational inference scheme from the probabilistic programming library Pyro v1.5.2^23^ to approximate the posterior distributions. Importantly, we ensured using simulations that assumptions we made for approximate posteriors are qualitatively good and that we can recover both parameters and planning depth with high accuracy. For this, we fixed model parameters to the inferred values and simulated behavioral responses for the same number of subjects. Using the same inference scheme, we again inferred posterior parameter distributions from these responses and validated that the true parameter values fall within the 95% credible interval of the estimates. Finally, we also validated that we can recover the actual planning depth on individual trials by ensuring that for at least 95% of posterior samples, the true planning depth had the highest probability.

### Statistical analysis

Having formalized the within-subject cognitive mechanism with a generative model (Eqs. 1–5), we then followed the standard procedure and next analyzed estimated parameters and additional between-subject variables with classical statistical tests. To analyze mean planning depths, we first calculated mean values for each subject and condition to compare groups and experimental conditions. To analyze the differences between groups in more detail, we set up a linear mixed effects model with random intercept and random slopes to test the effects of age group, noise and steps condition on planning depth. We also included condition-by-group interaction terms. In a second analysis, we tested to what extent mean planning depths can be explained by performance in tasks measuring fluid cognitive abilities. For this purpose, we aggregated subject-wise mean planning depths over the whole task and linearly regressed them on the performance outcomes of the IDP and the SWM as well as a group indicator. To investigate the role of planning time, we also included SAT reaction time as a predictor. A detailed description of the models can be found in the supplementary material (Eq. S20 ff.). As planning depth was the main focus of our study, we decided to use a simple two-sample *t*-test for group comparison of the remaining outcomes, i.e. SAT performance, reaction times, cognitive covariate performances and the remaining model parameters (alpha, beta, theta). As a measure of SAT performance, we calculated the achieved percentage of the maximum possible fuel score. If the normality assumption was not met, as indicated by a significant Shapiro–Wilk test statistic, we compared results with the non-parametric Mann–Whitney-U test. If variances were unequal, as indicated by Levene’s test, we checked results with Welch’s *t*-test. However, if there was hardly any difference in results of the alternative procedures compared to the standard *t*-test, we still report *t*-test statistics for better readability. To evaluate the quality of our computational model of participants’ choices in the SAT as described in the previous section, we analyzed how well the fitted model parameters explain variance in behavior. For this purpose, we linearly regressed SAT performance on all computational model parameters (mean planning depth, alpha, beta, theta).

All statistical analyses were carried out using IBM SPSS Statistics (Version 28) with a significance level of *α* = 0.05. For outlier analysis, we decided to exclude mini-blocks with an RT below 150 ms, which is a common timeframe for solely perceptual and motor processes^24^.

## Results

Descriptive statistics and group comparisons are depicted in Table 1. During outlier analysis, none of the SAT mini-blocks had to be excluded. However, there was one trial in the SAW and in the SWM with a reaction time below 150 ms that had to be excluded.

**Table 1:** Descriptive statistics and group comparison of model parameter and task outcomes.

	Younger adults (*N* = 25)	Older adults (*N* = 27)	*t*	*p*
**Sample characteristics**				
Age	26.4 (5.7)	68.8 (3.4)		
Gender (F/M)	7/18	13/14	2.23^b^	.136^b^
**Model parameters**				
Mean planning depth	2.1 (.3)	1.8 (.2)	4.14	< .001
Learning rate alpha	.0 (.1)	.0 (.0)	3.26	< .01
Inverse temperature beta	1.8 (.8)	1.1 (.4)	3.97	< .001
Response bias theta	− .2 (.3)	.2 (.6)	− 3.35	< .01
**Task performances (%)**				
Space adventure	57.7 (17.7)	38.2 (15.7)	4.22	< .001
Spot-a-word	62.2 (12.5)	69.9 (16.6)	− 2.47^a^	.013^a^
Spatial working memory	91.2 (7.3)	74.6 (22.1)	3.70	< .001
Identical pictures	65.7 (9.3)	45.8 ( 9.5)	7.62	< .001
**Task reaction times (s)**				
Space adventure	6.8 (3.9)	5.0 (2.6)	1.94	.058
Spot-a-word	5.5 (1.9)	5.7 (1.8)	− .41	.684
Spatial working memory	1.3 (.3)	1.7 (.4)	− 4.44	< .001
Identical pictures	2.4 (.3)	3.5 (.7)	− 7.66	< .001

Scores represent means and standard deviations (in parenthesis). For the Space Adventure Task, performance was defined as percentage of maximum possible points gained. For the other tasks, performance indicated percentage of correct trials.

^a^*Z-*statistic and corresponding asymptotic *p*-value based on non-parametric Mann–Whitney-U test as groups’ distributions deviated strongly from normality.

^b^Pearson’s chi-squared test statistic with one degree of freedom and corresponding *p*-value.

### Planning depth and performance

**Figure 3:**
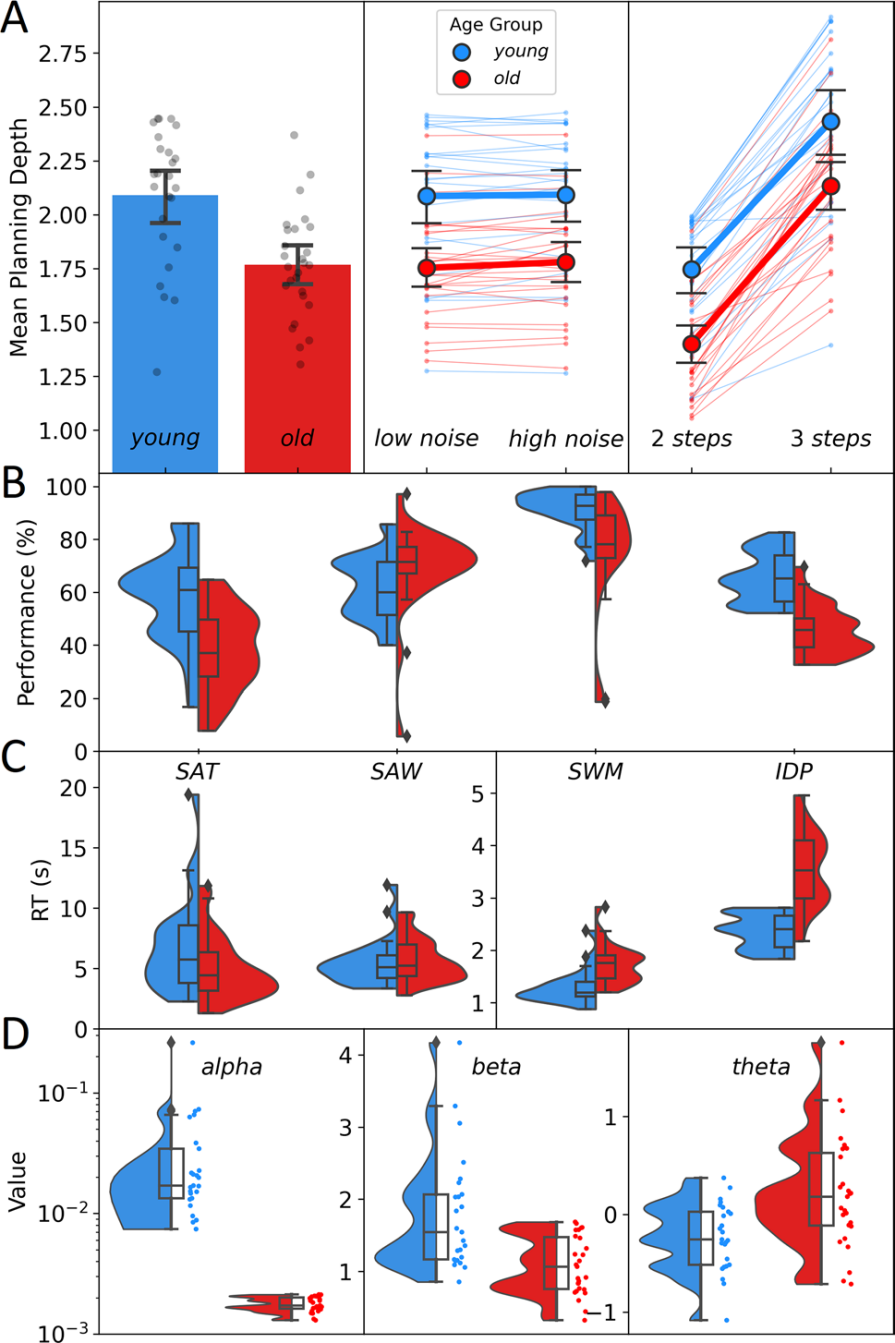
Mean Planning Depths, Task Outcomes and Model Parameters. (**A**) Mean planning depths in the Space Adventure Task (SAT) plotted for the whole task (left), over the two noise levels (middle) and numbers of steps (right). Small dots indicate mean values for individual participants. Large dots and bar plots represent group means. White small dots indicate boxplot medians. Error bars indicate 95% confidence intervals. (**B**) Density kernel estimate (DKE) plots and boxplots of subjects’ performance in the tasks. For the SAT, performance was defined as percentage of maximum possible points gained. For the Spot-a-Word Test (SAW), the Spatial Working Memory Task (SWM) and the Identical Pictures Task (IDP) performance indicated percentage of correct trials. (**C**) DKE plots and boxplots of reaction times for tasks with time limit (left) and without (right). (**D**) Raincloud plots of estimated model parameters at the subject-level: learning rate alpha, inverse temperature beta and response bias theta. Note that for alpha, the y-axis is transformed to the log-scale for better visualization of the densities.

The overall mean planning depth in older adults was approximately 0.3 steps lower compared to younger adults, see Fig. 3A. When taking into account intercept and noise in the linear mixed effects analysis (estimated parameters depicted in Table 2), this difference was statistically significant as indicated by the fixed group effect, *t*(53.90) = − 5.10, *p* < .001. Moreover, the number of steps significantly predicted mean planning depth, such that an increase in the number of actions that could be performed sequentially lead to deeper planning, *t*(52.14) = 14.93, *p* < .001. Although the steps effect showed considerable variation between subjects, there was a strong positive steps effect for almost all subjects. The effect of the noise condition was not significant, *t*(104.31) = .52, *p* = .607, i.e. participants did not change their planning depths when exposed to the condition with high uncertainty on jumps. This effect also did not show relevant between-subject variation. The interaction terms did not yield any significant effect for group*noise, *t*(104.43) = 1.16, *p* = .249, or group*steps, *t*(52.14) = .74, *p* = .465.

**Table 2:** Estimates of linear mixed effects model for mean planning depth*.*

	*b*	*SE b*	95% CI* b*		Random Effect (*η*)	*SE η*	95% CI *η*	
			*LL*	*UL*			*LL*	*UL*
Intercept	1.74	.05	1.64	1.84	.061	.012	.041	.090
Noise	.01	.01	− .02	.03	.000	.000	–	–
Steps	.69	.05	.59	.78	.049	.010	.032	.074
Group	− .36	.07	− .50	− .22	–	–	–	–
Group*Noise	.02	.02	− .01	.06	–	–	–	–
Group*Steps	.05	.06	− .08	.18	–	–	–	–

The covariance parameter for Noise was redundant as interindividual variance was too small. The confidence interval could not be computed by SPSS. CI = confidence interval; *LL* = lower limit; *UL* = upper limit.

Performance (percentage of maximum possible points gained) was significantly lower for older adults, *t*(50) = 4.22, *p* < .001 (see Fig. 3B). There was no significant difference in mean reaction times between groups, only a visual trend of tendentially faster mean reaction times for older adults, *t*(50) = 1.94, *p* = .058 (see Fig. 3C). Across conditions and groups, longer reaction times in the SAT were associated with deeper mean planning depths (*r* = .512, *p* < .001) and higher SAT performance (*r* = .624, *p* < .001). SAT performance and planning depth also showed a significant positive correlation (*r* = .861, *p* < .001).

### Cognitive covariates

Older adults showed a significantly lower performance in the tasks associated with indicators of fluid intelligence, i.e. the SWM (*t*(32.06) = 3.70, *p* < .001) and the IDP (*t*(50) = 7.62, *p* < .001, see  Fig. 3B). A correlation analysis revealed significant associations of mean planning depth with IDP performance (*r* = .37, *p* < .01) and SWM performance (*r* = .32, *p* < .05). Including these covariates in a linear regression model of subject-wise mean planning depths (see Table 3 for parameter estimates) did not yield any significant effect for these predictors. Controlling for these covariates as well as speed in the SAT, the group variable still indicates a significant difference in planning depth between groups.

**Table 3:** Linear regression analysis.

Predictor	Unstandardized coefficients		Standardized coefficients	*t*	*p*	95% CI *b*	
	*b*	*SE*	*Beta*			*LL*	*UL*
Intercept	1.80	.28		6.309	< .001	1.12	2.37
Group	− .25	.11	− .40	− 2.367	.022	− .47	− .04
IDP_PER (%)	.00	.00	− .02	− .116	.908	− .01	.01
SWM_PER (%)	.00	.00	.04	.306	.761	.00	.01
SAT_RT (s)	.04	.01	.40	3.356	.002	.02	.06

CI = confidence interval; *LL* = lower limit; *UL* = upper limit.

Model Summary: *R*^*2*^ = .410; Adj. *R*^*2*^ = .360; *SE* = .258.

### Model parameters

We found that values for learning rate $\alpha$ were overall close to zero suggesting stable beliefs about transitions uncertainties during the experiments. Learning rates for older adults were significantly lower compared to younger adults, *t* = 3.26, *p* ≤ .01 (see Fig. 3 D). Older adults showed a significantly lower inverse temperature $\beta$, *t*(34.74) = 3.97, *p* < .001 indicating higher response noise. The response bias $\theta$ differed significantly between groups, *t*(50) = − 3.35, *p* < .01. This means that younger adults showed a significant response bias towards the deterministic "move" action indicated by a negative theta value, *t*(24) = − 3.37, *p* < .01, whereas older adults showed no bias significantly different from zero, *t*(26) = 1.92, *p* = .066.

All model parameters (mean planning depth, $\alpha$, $\beta$, $\theta$) explained 88.2% of the variance in SAT performance (*R*^2^ = .882) with a significant contribution of mean planning depth (*Beta* = .528, *p* < .001) and $\beta$ (*Beta* = .500, *p* < .001). Detailed results of the underlying linear regression can be found in the supplementary material.

## Discussion

In this study, we aimed at assessing how planning depth is modulated by old age. We found that in our sequential decision-making task that required forward planning in order to maximize outcomes, performance was lower in older adults compared to younger adults, i.e. they collected less points. Using a model-based analysis, we found in older adults lower inferred planning depths and higher response noise. Notably, older adults also showed substantially lower performance in cognitive tasks measuring processing speed, working memory and in our forward planning task slightly (trend-wise significant) faster reaction times. Even when controlling for lower processing speed, spatial working memory and reaction times, we still found the robust result of lower planning depth in older adults.

Our main finding of reduced planning depth in older adults is in line with established findings of cognitive aging^17^ and evidence from classical planning tasks^8^. Moreover, planning depth of both groups increased with task complexity in the form of a deeper decision tree (2-steps to 3-steps condition) while the difference between age groups did not change (group*steps interaction). Interestingly, in the 3-steps condition most subjects, on average, planned ahead 2 or more steps, but planned ahead less than 2 steps in in the easier 2-steps condition. This indicates that in the SAT, not only the ability to plan but also invested effort plays a crucial role. This assumption is well in line with recent proposals of how cognitive control is regulated such as the Expected Value of Control (EVC) framework or accounts of computational rationality^25,26^: if people plan in a resource-rational manner, they should balance the costs and benefits of investing cognitive resources to plan deeper. On the cost side, forward planning was probably more demanding for older adults because they had to compensate for more limited cognitive resources. On the benefit side, evidence from neuroimaging suggests that reward sensitivity is shifted from monetary reward to social reward in old age^27^. Because participants in the SAT were rewarded with a performance dependent amount of money, the benefits of planning might have been lower for older adults. Therefore, the expected value of control/planning was probably reduced in older adults which might have led to lower motivation and planning depth, or the use of simpler, less costly heuristical forward planning algorithms^20^. This would also explain why older adults reacted faster in the SAT in our sample and the association of RT and planning depth in our study. RT is a well-known indicator of cognitive cost of the underlying computations^28^. Nevertheless, the group differences in planning depth remained significant when controlling for reaction time. This indicates that additional differences are at play and that group differences are not just due to a difference in trading off planning depth and speed. Explicitly modelling such hypothetical alternative planning algorithms in future model comparison studies of forward planning could provide further insight.

Differences in effort allocation between both age groups might also explain why neither spatial working memory nor processing speed were significant predictors of mean planning depth. The tasks used to measure general cognitive performance are not reward-based but motivate participants with time pressure. Therefore, weighing up effort and reward might play a minor role. In the SAT however, we assume that especially in the 2-steps condition individual limits of storage were mostly not reached due to reduced effort and reduced allocation of cognitive resources.

A second explanation of lower task performance suggests that older adults, although extensively trained, build noisier task representations^29–31^. This effect could also underlie lower inferred planning depth. For all applied RL agent models, correct task knowledge was assumed but older adults might have forgotten aspects of the task during the course of the experiment. Noisy or erroneous task representations with still extensive planning could result in similar choices as lower planning depth with correct task models. It is even conceivable that these mechanisms are intertwined: noise in representations could accumulate during forward planning computations and lead to less precise outcome predictions with deeper planning, which would effectively result in a gradual limitation of planning depth. Another line of argument from Jiang et al.^32^ elaborates further. These authors show mathematically that in the case of incorrect task models, limitation of planning depth can even be beneficial as it can avoid overfitting in policy selection. In other words, they show that it is useful not to set up far-reaching plans on imprecise knowledge. Transferred to the SAT, this means that participants might continuously estimate the uncertainty of their belief over the task structure, e.g. the travel pattern or transition probabilities. This uncertainty is presumably higher in older adults and therefore they might have dynamically adapted their planning horizons. Unfortunately, the set of possible incorrect or noisy task representations is extremely large making exact inference practically infeasible. In the context of the present study, deviations from correct task representations might therefore instead partly be reflected in lower inverse temperature values, which was the case for older adults.

A third explanation for reduced task performance is that group differences could also have been driven by processes independent from the computation of the prediction, e.g. by constantly increased response noise. The latter is well captured by the inverse temperature parameter, which indeed was decreased in older compared to younger adults. This could explain why we only found main effects of age group and task complexity but no group*steps interaction effect. Moreover, this parameter was—besides planning depth—the best predictor of performance in the SAT, clearly indicating a central influence of response noise on outcomes of forward planning. Though higher response noise well accounts for lower task performance (more random choices), it does not explain lower planning depth, since both parameters of the computational model are theoretically independent.

### Limitations

Contra-intuitively, inferred planning depth was almost equal between the high and low noise condition for both groups. This is inconsistent with a previous study that found decreased model-basedness when state transitions were less predictable in the two-step task^33^. Post-hoc simulations revealed that this result was most likely caused by a suboptimal design of the task: high noise mini-blocks yielded on average higher rewards and were presented at a later stage during the experiment. Thus, motivation and learning effects are potential confounding variables for the noise effect. We therefore refrain from any interpretations here and will instead work on an improved version to address this in the future.

Furthermore, we cannot exclude that older adults might have forgotten aspects of the task during the course of the experiment. For future studies, we therefore aim at using a simplified version of the task to minimize this potential confound.

There are two other potential limitations of the current work that we wish to address: first, the cross-sectional design of our study did not control for systematic differences in computer (game) experience between the two cohorts. This could be a significant confounder of the age effect which should be considered in further investigations, e.g. by longitudinal studies or by attempts of measuring the amount of computer experience directly.

Second, we propose a limitation of planning depth as a mechanism to keep model-based control affordable and our computational model showed good validity as the model parameters could explain most of the variance in planning performance. However, we want to highlight that our model just captures one example out of a variety of possible planning algorithms and that humans most likely apply multiple strategies^20,29^, which seems to be especially relevant for older adults. This is also indicated by the inverse temperature parameter which—besides response noise—can also capture mismatch between model and data. Higher response noise (lower inverse temperature) in older adults could therefore also point towards older adults using alternative heuristical strategies which are not covered by our model.

## Conclusion

Taken together, our findings strongly point towards an age-related reduction of forward planning that cannot be explained by reduced cognitive abilities (i.e. working memory or processing speed) in older adults. Rather, reduced task performance in older adults seems to be partly due to higher randomness of their choices (higher response noise) and reduced depth of forward planning. We speculate that the reduction of planning depth in older adults is driven by reduced allocation of model-based effort, with older adults seemingly applying lower-cost strategies. Noisier task representations might also play a role in this process. We assume that additional strategic differences are at play and that group differences in planning depth are not just due to differences in trading off costs and benefits of planning. Explicitly modelling and testing these hypothetical alternative planning algorithms in future studies of forward planning could shed more light on this issue.

## Supplementary Information

Supplementary Information.

## Data Availability

All data generated or analysed during this study along with the code and scripts necessary to perform the model-based inference and statistical analyses are available in the plandepth_age Github repository, https://github.com/jeffensen/plandepth_age.
