# Supplementary material for: Shorter planning depth and higher response noise during sequential decision-making in old age
Source: Sci Rep. 2023 May 11;13:7692. doi: 10.1038/s41598-023-33274-0 (PMC10175280; doi:10.1038/s41598-023-33274-0)
Supplement: Supplementary file 1 — Supplementary Information. [file 41598_2023_33274_MOESM1_ESM.pdf]

Shorter Planning Depth and Higher Response Noise During Sequential Decision-Making in Old Age

Johannes Steffen[1], Dimitrije Marković[2], Franka Glöckner[2], Philipp T. Neukam[1,3], Stefan J. Kiebel[2], Shu-Chen Li[2], Michael N. Smolka[1*]

[1]*Department of Psychiatry and Psychotherapy, Technische Universität Dresden, Dresden, Germany*

[2]*Department of Psychology, Technische Universität Dresden, Dresden, Germany*

[3]*Department of Psychiatry, Icahn School of Medicine at Mount Sinai, New York, NY, USA*

[*]*Corresponding author*

Supplementary Material

Neurocognitive Tasks

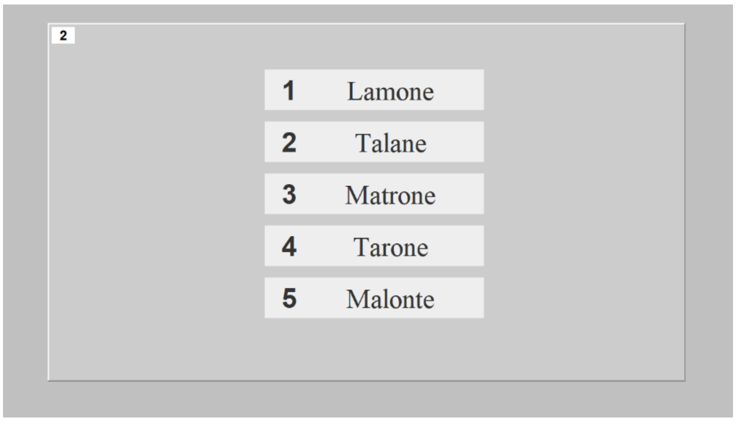

Figure S1 *Example trial of the Spot-a-word Test*. In each trial, 5 items were presented containing 4 pseudowords and one existing German noun. Participants were instructed to indicate the real word by pressing the corresponding number on the keyboard (1-5) as quickly and accurately as possible although there was no time limit. The performance was calculated as percentage of correct responses relative to the total of 35 trials.

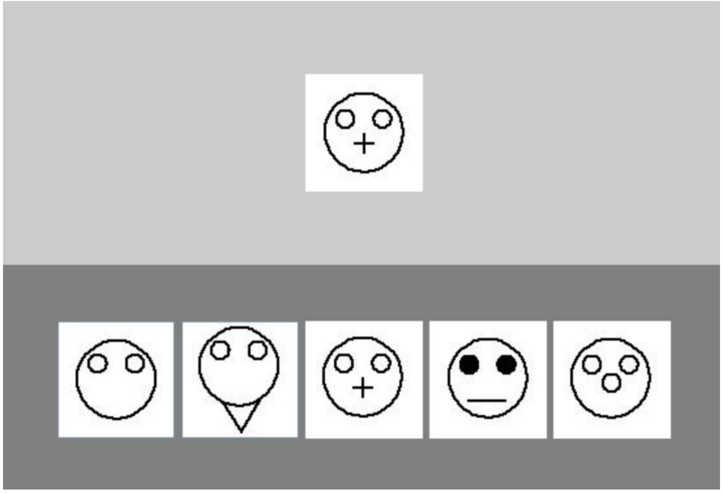

Figure S2 *Example trial of the Identical Pictures Task*. Participants were instructed to find out of 5 symbols the stimulus matching the target stimulus presented at the top by pressing the corresponding number on the keyboard (1-5) as quickly and accurately as possible. The task had an overall time limit of 80 seconds and performance was calculated as percentage of correct responses relative to the total of 46 trials.

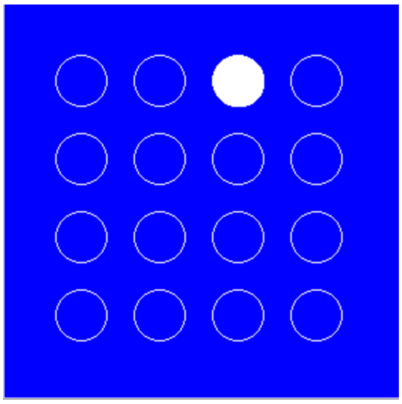

Figure S3 *Example stimulus of the Spatial Working Memory Task*. Participants were presented with a 4 x 4 grid of circles in which a series of dots were displayed consecutively in specific locations of the grid. At the end of each sequence, one circle was marked and participants had to indicate whether a dot was presented at that position or not (i.e., location memory condition). If they affirmed, a digit was shown at the marked position and participants then had to decide whether the dot was presented in that serial position or not (i.e., sequence memory condition). Working memory load was varied by dot sequence length either being 4 or 7. (i.e., working memory load levels). The time limit for each trial was 5 seconds for both conditions. As data for the sequence memory condition was very limited, we only used the location memory data for analysis and calculated performance as percentage of correct responses relative to the total of 96 trials across both load level.

Characteristics of the Space Adventure Task

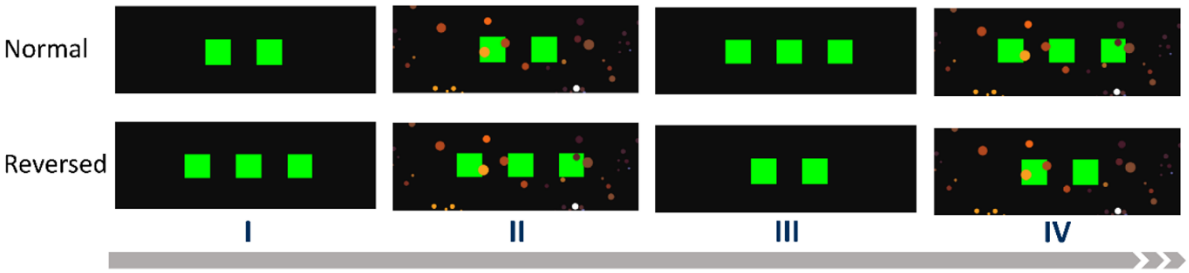

Figure S4 *Schematic illustration of counterbalancing in the Space Adventure Task*. Each participant had to solve 100 mini-blocks with varying number of actions (two-step and three-step) indicated by the number of green boxes and varying level of uncertainty for the transition of the jump action where high uncertainty was indicated by small asteroids. Participants experienced these conditions in a blocked manner, i.e. the experiment was divided into four phases of 25 mini-blocks with equal number of steps and noise level indicated by the roman numerals. The order of the steps condition was counterbalanced between subjects, i.e. half of them started with two-step mini-blocks ('Normal' order) while the other half started in 'Reversed' order with three-step mini-blocks first.

Training Procedure of the Space Adventure Task

As task comprehension and training is of particular concern in aging research, we designed an extensive training for the Space Adventure Task with instructions as self-explaining as possible. During the training, explicit task knowledge was assessed by two tests (see 7. and 16. below). Every participant passed these tests. Moreover, an examiner was present and offered help for any questions throughout the training and the experiment. As there was no time limit, subjects could receive all support they needed. The overall procedure of the training is listed below. Details on the implementation can be found in public repository of the task: https://github.com/dimarkov/sat.

Instructions

1. Introduction to main goal: maximize points/fuel
2. Introduction to planet systems and rocket
3. Introduction to two actions, respective keys and action costs
4. Introduction to fuel bar at the top
5. Explanation of move action and trying it out to familiarize (each starting position twice)
6. Explanation of and trying out jump action:
    a. Instruction to memorize target planets very well and trying it out to familiarize (each starting position twice)
    b. Presentation of travel pattern image and instruction to memorize it very well without time limit (as it was not available afterwards, especially not during the run of the experiment)
7. Travel pattern test:
    - Subjects had to input the target planet number for a given starting configuration (each position was tested three times, making 18 test trials in total).
    - If subjects made more than one mistake during the test, they underwent a personal tutoring by the examiner until they could recall every target planet correctly
8. Explanation of probabilistic nature of jump action
9. Explanation of high and low noise planet systems with/without asteroids respectively
10. Trying out jumping in low noise condition starting from each position twice; implicit learning of low transition noise (p = 0.1) by experiencing a miss in 1 out of 12 jumps
11. Trying out jumping in high noise condition starting from each position twice; implicit learning of high transition noise (p = 0.5) by experiencing a miss in 6 out of 12 jumps

(Note that here, participants were only required to learn that one probability (low noise) was sufficiently larger than the other (high noise), in order to pass the optimal planning test and perform the task. The choice measure of the task was not very sensitive to exact beliefs about transition probabilities)

12. Introduction to planet types with resp. rewards and instruction to memorize them well
13. Introduction to limited number of actions
14. Summary
15. Explanation that forward planning is beneficial with a detailed example
16. Optimal Planning Test:

Subjects were presented 4 example mini-blocks and received feedback if they found the optimal flight route. Each mini-block was repeated until the subject's response was optimal.

Practice

If subjects had no open questions, they performed 20 practice mini-blocks without feedback on optimality. The set of practice mini-blocks contained 5 mini-blocks per condition (2 or 3 steps and low or high noise)

Main Experiment

If subjects had no open questions and there was no lack of task knowledge apparent during practice, subjects performed the 100 mini-blocks used for subsequent analysis with 25 mini-blocks per condition (see ***Figure S4***).

The Value Iteration Algorithm

The value iteration algorithm [1]is a dynamic programming algorithm for finding the optimal policy π (a policy is a state-action mapping and can be considered to be the plan how to act in the current and possible future states) in a fully observable Markov decision process. Each mini-block of the Space Adventure Task is such a Markov decision process with a finite horizon of two or three steps depending on the experimental condition. A mini-block can thus be defined as a tuple:

$$\big(T, S, R, r(s), A, C, c(a), p(s_{t+1}|s_t, a_t)\big), \qquad \text{(S1)}$$

where

- T denotes the number of trials for the mini-block (the horizon or step size) with: $T = \begin{cases} 2, & \text{for two-step mini-blocks,} \\ 3, & \text{for three-step mini-blocks,} \end{cases}$ hence $t = 1, \ldots, T$.
- $S = \{0, \ldots, 5\}$ denotes the set of task states, i.e. the six positions of the planet configuration.
- $R = \{-20, -10, 0, 10, 20\}$ denotes the set of rewards.
- $r(s)$ denotes the immediate reward in each state, i.e. the planet type in each position of the planet configuration (this is equivalent to the reward probability $p(r|s)$ if reward would be given probabilistically).
- $A = \{0,1\}$ denotes the set of possible actions, where 0 corresponds to moving clockwise and 1 to jumping.
- $C = \{-2, -5\}$ denotes the set of action costs where jumping has higher costs.
- $c(a)$ denotes the immediate costs of executing action a.
- $p(s_{t+1}|s_t, a_t)$ denotes the transition probability, i.e. the probability to reach a subsequent state s_{t+1} from a given state s_t by executing action a_t.

The value iteration algorithm can be thought of as solving the problem of finding the optimal action sequence by breaking it down into single decision problems for each planning step and going backwards through the chain of decisions thereby reducing the exponential complexity to not more than polynomial complexity [1], as illustrated in ***Figure S5***.

Concretely, the procedure starts with considering the last action decision to be made for all possible states and choosing the best action for being in each of these states. This decision yields the optimal value (maximum expected value) for each state in that period, i.e. the state-value $V(s, d)$, with initial planning depth $d = 1$. In the following iteration, the case of being in the next-to-last step is considered for all the possible states. Once again, the best action is chosen, given that the optimal values of being in various states at the next time period is now known. This yields new optimal state-values for $d = 2$. This process is continued until the present step or the maximum planning depth is reached.

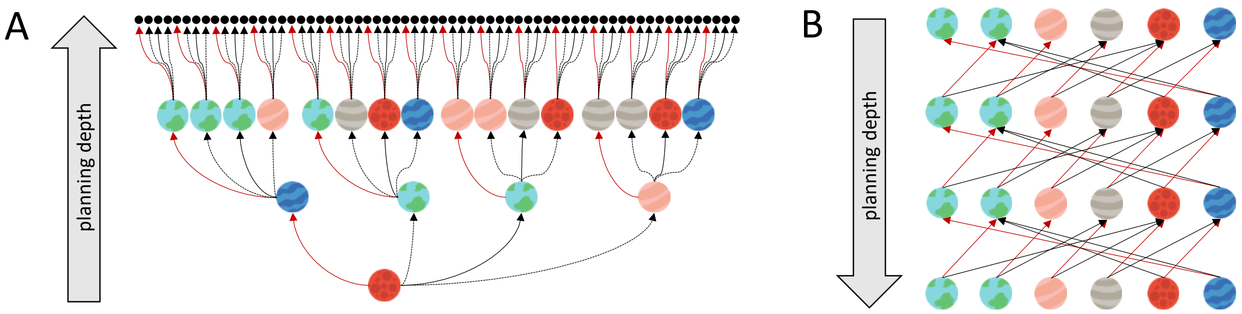

Figure S5 *Illustration of Forward Planning Complexity*. This figure illustrates the forward planning complexity of exhaustive search (A) versus value iteration (B) by taking the example of the mini-block depicted in Figure 1 B. Red and black arrows indicate moves and jumps, respectively. Dashed lines represent transition failures of the probabilistic jump action (omitted in B for better readability).

The value iteration algorithm with planning depth $d \epsilon \{1,2,3\}$ is mathematically defined by:

$$Q(s_t, a_t, d) = \sum_{s_{t+1}} p(s_{t+1}|s_t, a_t)[c(a_t) + r(s_{t+1}) + \gamma V(s_{t+1}, d)] \quad \text{(S2)}$$

$$V(s_t, d) = \begin{cases} 0 & , for\ t > min(T, d), otherwise \\ \max_{a_t} Q(s_t, a_t, d) & \end{cases} \quad \text{(S3)}$$

Here, $Q(s, a)$ denotes state-action values and $V(s_t, d)$ state-values. Setting $V(s_t, d)$ to zero serves as the starting point of the backward induction. This is the case when either the planning depth d or the number of steps T is reached. The parameter γ denotes the discounting rate of future outcomes. Note that we considered $\gamma = 1$ as this parameter is not simultaneously identifiable with planning depth. For some forward planning algorithms in machine learning, γ is explicitly transformed to limited planning depth, e.g. by $d = \frac{1}{1-\gamma}$, [2].

Parameter Inference

For inference, we used a hierarchical generative model, which states that for each model parameter alpha, beta, and theta there is a global hyper-prior that defines prior mean and variance for each parameter. The hierarchical structure helps pulling the information between participants, and constraining possible ranges for each of the model parameters, as data obtained from individual participants is not sufficient to identify a constrained posterior for all parameters. Note that the group level hyper-prior $p(G|\lambda)$ captures both prior mean and prior uncertainty on the level below, hence $G = (g_1, g_2, g_3, \sigma_1, \sigma_2, \sigma_3)$ for which we use a product of normal-inverse gamma distributions as a prior (see eq. S8). Concretely, for the inference of free model parameters $\gamma = (g_1, g_2, g_3) = (logit^{-1}(\alpha), ln(\beta), \theta)$ and the trial dependent planning depth d_t, we used the following hierarchical generative model:

$$p(G|\lambda) \prod_{n=1}^{N} p(\gamma_n|G) p(r_n|\alpha) \prod_{b=1}^{100} p\left(a_b^n \middle| s_b^n, r_{n,f(b)}, \gamma_n\right), \qquad \text{(S4)}$$

where n denotes participant's number, b the mini-block, G the group level hyper-priors and $\phi = (\lambda, \alpha_1, \alpha_2, \alpha_3, \alpha_4)$ the set of hyper-parameters (see below). $r_{n,f(b)}$ denotes the participant specific prior over a planning depth, dependent on the current mini-block, that is, the maximum number of trials of the experiment. The function $f(b) \in \{1,2,3,4\}$ maps the current mini-block to corresponding value dependent on the phase of the experiment, hence:

$$f(b) = \begin{cases} 1, for\ T = 2 \\ 2, for\ T = 2 \wedge noisy \\ 3, for\ T = 3 \\ 4, for\ T = 3 \wedge noisy \end{cases} \qquad \text{(S5)}$$

Note that the participants specific prior over planning depth $r_{n,f(b)}$ specifies the probability that participants use planning depth d at each trial of phase $f(b)$ of the experiment; where $p\left(d_{b,t=1}^n = d \middle| r_{n,f(b)}\right) = r_{n,f(b),d}$ and $\sum_d r_{n,f(b),d} = 1$. The probability over planning depth specifies model likelihood (probability of generating action a) in the from of a mixture distribution as:

$$p\left(a_b^n \middle| s_b^n, r_{n,f(b)}, \gamma_n\right) = \sum p\left(d_{b,n} = d \middle| r_{n,f(b)}\right) p(a_b^n | s_b^n, d_b^n, \gamma_n) \qquad \text{(S6)}$$

To infer the posterior over free model parameters, both on the group level and on the subject level, we used stochastic variational inference (for more details on the approach we refer the reader to one of the recent introductory reviews[3]). In stochastic variational inference one minimizes the variational free energy (loss function) with respect to the parameters of the approximate posterior q using stochastic gradients estimated via samples from the approximate posterior. In what follows we describe the key components of the process.

Given the generative model, we approximate the posterior using the following factorisation of the true posterior:

$$P(G, \gamma_{1:N}, r_{1:N} | A_n, S_n, \phi) \approx q(G) \prod_{n=1}^{N} q(\gamma_n) \prod q\left(r_{n,i}\right) \quad \text{(S7)}$$

where A_n, S_n denote set of all responses and states (stimuli) of the nth participant.

a) Hierarchical Prior Distribution

The group level hyper-prior over γ_n was defined as a Normal—InverseGamma distribution for each free parameter:

$$p(G|\lambda) = \prod_{k=1}^{3} N\,\Gamma^{-1}(g_k, \sigma_k^2; m_k, l_k, w_k, v_k) \quad \text{(S8)}$$

where m, l, w, v denote parameters of the Normal—InverseGamma distribution; thus, $\lambda = (m, l, w, v)$. The role of the group level prior is to pull together estimates over different subjects, hence improve estimates of model parameters which carry high posterior uncertainty on the level of single subjects. The subject level prior was defined as a (multivariate) normal distribution:

$$p(\gamma_n|G) = \prod_{k=1}^{3} N\left(\gamma_{n,k}; g_k, \sigma_k^2\right). \quad \text{(S9)}$$

Finally, the prior over planning depth corresponded to a Dirichlet distribution, where

$$p(r_n|\alpha) = \prod_{i=1}^{4} Dir\left(r_{n,i}|\alpha_i\right) \quad \text{(S10)}$$

where i denotes different phases of the experiment.

b) Variational Approximation

The approximate posterior q was factorised as follows:

$$q(G, \gamma_{1:n}, r_{1:n,1:4}) = q(G) \prod_{n=1}^{N} q(\gamma_n) \prod_{i=1}^{4} q(r_{n,i}), \quad \text{(S11)}$$

where

$$q(G) = q(g_1, g_2, g_3, \sigma_1^2, \sigma_2^2, \sigma_3^2) = \frac{1}{8\sigma_1^2\sigma_2^2\sigma_3^2} N_6(\mu, \Sigma) \quad \text{(S12)}$$

$$q(\gamma_n) = N_3(m_n, Y_n) \quad \text{(S13)}$$

and

$$q(r_{n,i}) = Dir(\alpha'_{n,i}) \quad \text{(S14)}$$

The approximate posterior estimate of relevant model parameters is obtained as the minimiser of the variational free energy, with respect to the sufficient statistics of the factorised approximate posterior $\psi = (\mu, \Sigma, m_{1:N}, Y_{1:N}, \alpha'_{1:N,1:2})$, where we at the same time optimise the generative model with respect to the hyper-parameters ϕ, effectively performing an empirical Bayes estimate with respect to hyper-parameters. The whole optimisation procedure rests upon the stochastic variational inference scheme implemented in Pyro [4] (v1.5.2), a Python based probabilistic programming library built on PyTorch (more details on this can be found at https://github.com/pyro-ppl/pyro).

c) Stochastic variational inference

Starting with the definition of the variational free energy

$$F(\psi, \phi) = E_q \left[ln \frac{q(G, \gamma_{1:N}, r_{1:N,1:4} | \psi)}{P(A_{1:N}, G, \gamma_{1:N}, r_{1:N,1:4} | S_{1:N}, \phi)} \right] \quad \text{(S15)}$$

we obtain the minimum of the free energy with respect to parameter sets (ψ, ϕ) using stochastic gradient descent. Hence, for K samples from the approximate posterior Q the stochastic gradient steps are obtained as

$$\Delta\phi = \eta_t^{\phi} \frac{1}{K} \sum_{k=1}^{K} \nabla_{\phi} P\left(A_{1:N}, G^k, \gamma_{1:N}^k, r_{1:N,1:4}^k \middle| S_{1:N}, \phi\right) \tag{S16}$$

for optimising free energy with respect to the parameters of the generative model (ϕ), and

$$\Delta\psi = -\eta_t^{\psi} \frac{1}{K} \sum_{k=1}^{K} \left(ln \frac{q\left(G^k, \gamma_{1:N}^k, r_{1:N,1:4}^k \middle| \psi\right)}{P\left(A_{1:N}, G^k, \gamma_{1:N}^k, r_{1:N,1:4}^k \middle| S_{1:N}, \phi\right)} + 1 \right) \nabla_{\psi}\, ln\, q\left(G^k, \gamma_{1:N}^k, r_{1:N,1:4}^k \middle| \psi\right) \tag{S17}$$

for minimizing free energy with respect to the parameters of the approximate posterior (φ). In practice, we use the Adam optimizer for estimating the direction of the stochastic gradient for individual parameters in the two sets. More details on this can be found at https://pyro.ai/examples/svi_part_iii.html.

d) Planning Depth Posterior

The above stochastic optimization scheme allows us to infer the posterior over parameters on the group level $q(G)$, and on the subject specific level $q(\gamma_n, r_n)$. However, the quantity of interest which is the subject specific posterior over planning depth $q(d_b^n)$ is still missing, as we have marginalized over the planning depth inside the likelihood function (see eq. (5) in the main text and eq. S6). Hence here we describe the steps we used to recover the marginal posterior over planning depth.

We obtain the conditional posterior over planning depth (at trial $t = 1$ of a mini-block b) as

$$q\left(d_b^n \middle| \gamma_n, r_{n,f(b)}\right) = \frac{p\left(a_{1,b}^n \middle| s_{1,b}^n, d_b^n, \gamma_n\right) p\left(d_b^n \middle| r_{n,f(b)}\right)}{p\left(a_{1,b}^n \middle| s_{1,b}^n, r_{n,f(b)}, \gamma_n\right)} \tag{S18}$$

Hence for a given approximate marginal posterior distribution $q(\gamma_n, r_n) = q(\gamma_n) \prod_{i=1}^{4} q\left(r_{n,i}\right)$, we obtain the marginal (approximate) posterior over planning depths as:

$$q(d_b^n) = \frac{1}{K} \sum_{k=1}^{K} \frac{p\left(a_{1,b}^n \middle| s_{1,b}^n, d_{1,b}^n, \gamma_n^k\right) p\left(d_b^n \middle| r_{n,f(b)}^k\right)}{p\left(a_{1,b}^n \middle| s_{1,b}^n, r_{n,f(b)}^k, \gamma_n^k\right)}, \tag{S19}$$

where K denotes the number of samples from the marginal posterior distribution $q(\gamma_n, r_n)$ of the nth participant.

Note: Although we perform posterior estimate over planning depth and obtain an approximate posterior distribution $q(d_b^n)$ for each block and participant, the uncertainty of posterior estimates is typically small, as we observed well pronounced modes. This small uncertainty

becomes negligible once we average over 25 mini-blocks to obtain expected planning depth within a phase of the experiment. The main source of uncertainty for the average planning depth then becomes within-subject behavioral variability from one mini-block to the next.

Data Analysis

Linear Mixed Effects Model of Mean Planning Depth

We analysed mean planning depths with a linear mixed effects (LME) model. The analysis was performed using the MIXED command of the software SPSS (version 28) with an alpha level of 0.05. The model was formulated as follows:

$$\begin{aligned} Y_{ij} = b_{00} + b_{01} * group_j + b_{10} * noise_{ij} + b_{20} * steps_{ij} + b_{30} \\ * group * noise_{ij} + b_{40} * group * steps_{ij} + \eta_j^{b_{00}} \\ + \eta_j^{b_{10}} + \eta_j^{b_{20}} + r_{ij} \end{aligned} \quad \text{(S20)}$$

Here, the mean planning depth of participant j at condition i (2/3 steps and low/high noise) is indicated as Y_{ij}, respectively. The model includes an intercept as well as the effect of age as indicated by the group indicator which was set to 0 for younger adults and 1 for older adults. On the condition level, the model includes effects for the noise level and number of steps. Furthermore, we included condition-by-group interaction terms. All these fixed effects are depicted by the corresponding coefficient b; r_{ij} represents the error term. Individual variation of effects around the fixed effect, i.e., random effects, are depicted by η_j, i.e. the random intercept and random slopes for the effects of the noise and steps condition. As covariance structure we used the variance components option assuming no correlation between the estimated covariance parameters, i.e. random effects. Fitting the model with minimal assumptions (unstructured covariance matrix) did not significantly improve the model fit. To conduct hypothesis tests for the fixed effects, we used standardized parameter estimates (Estimate / $SE_{Estimate}$) which follow a t-distribution with approximated degrees of freedom [5].

Model	Fixed Effects	Random Effects	Number of Parameters	Covariance Structure	-2 LL	AIC
1	6	3	10	Variance Components	-203.175	-183.175

Table S1 *LME Model Summary*

Parameter	Estimate	*SE*	*df*	*t*	*p*	95% CI	
						LL	*UL*
Intercept	1.74	.05	53.90	34.52	<.001	1.628	1.836
Group	-.36	.07	53.90	-5.09	<.001	-.464	-.176
Noise	.01	.01	104.43	.52	.932	-.025	.023
Steps	.69	.05	52.14	14.93	<.001	.573	.782
Group*Noise	.02	.02	104.43	1.16	.249	-.015	.057
Group*Steps	.05	.06	52.14	.74	.465	-.081	.175

Table S2 *Estimates of Fixed Effects.* CI = confidence interval; LL = lower limit; UL = upper limit.

Parameter	Estimate	*SE*	Wald *Z*	*p*	95% CI	
					LL	*UL*
Residual	.004	.001	7.23	<.001	.003	.006
Intercept	.061	.012	4.93	<.001	.041	.090
Noise	.000	.000	-	-	-	-
Steps	.049	.010	4.69	<.001	.032	.074

Table S3 *Estimates of Covariance Parameters*. The covariance parameter for Noise was redundant as interindividual variance was too small. The test statistic and confidence interval could not be computed by SPSS. CI = confidence interval; *LL* = lower limit; *UL* = upper limit.

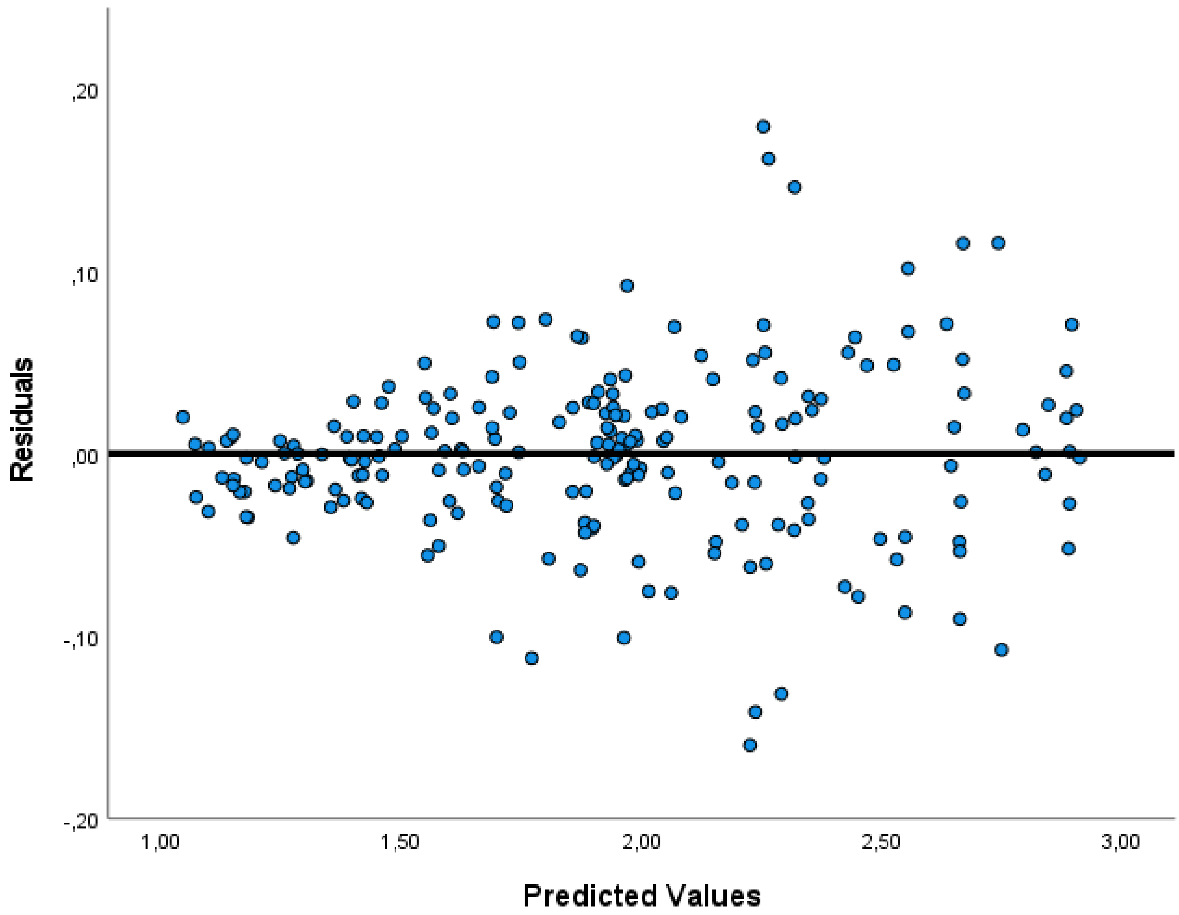

Figure S6 *LME Model Residual Plot.* Scatterplot of residuals against predictive values of the linear mixed effects (LME) model of mean planning depth. The plot shows slightly higher residuals variance for higher predicted values. This is expected due to the larger range of possible values for mean planning depth in the 3-step condition which should not violate the assumption of homoscedasticity.

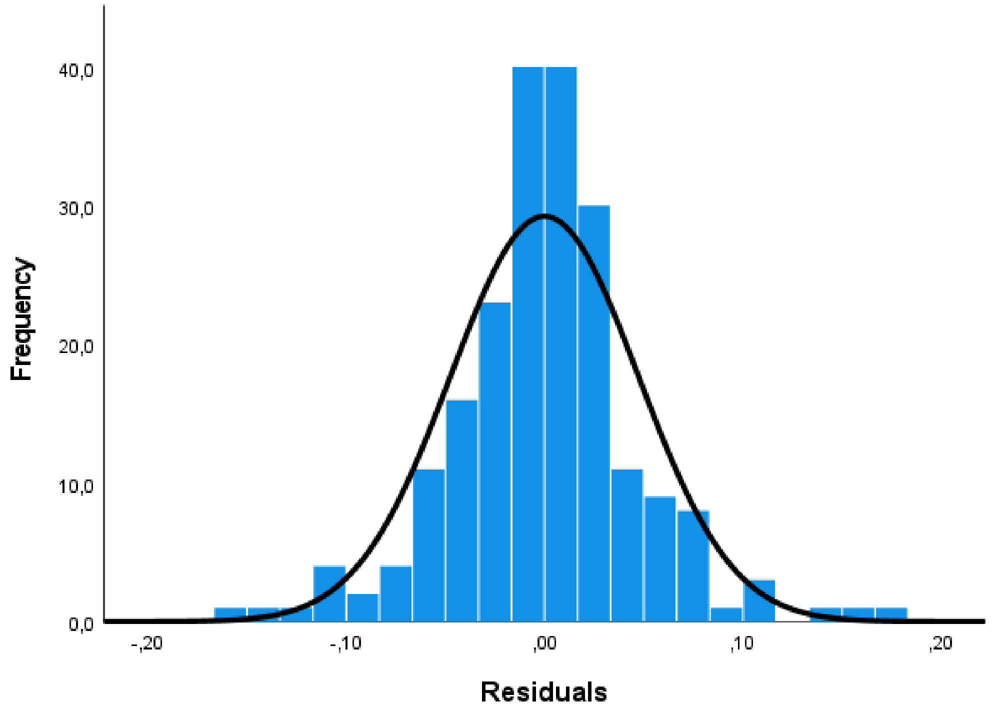

Figure S7 *LME Model Histogram of Residuals.* The plots shows the histogram of residuals of the linear mixed effects (LME) model of mean planning depth. The plot shows all residuals, i.e. for both groups and a fitted normal curve in black. Overall, residuals seem to be normally distributed.

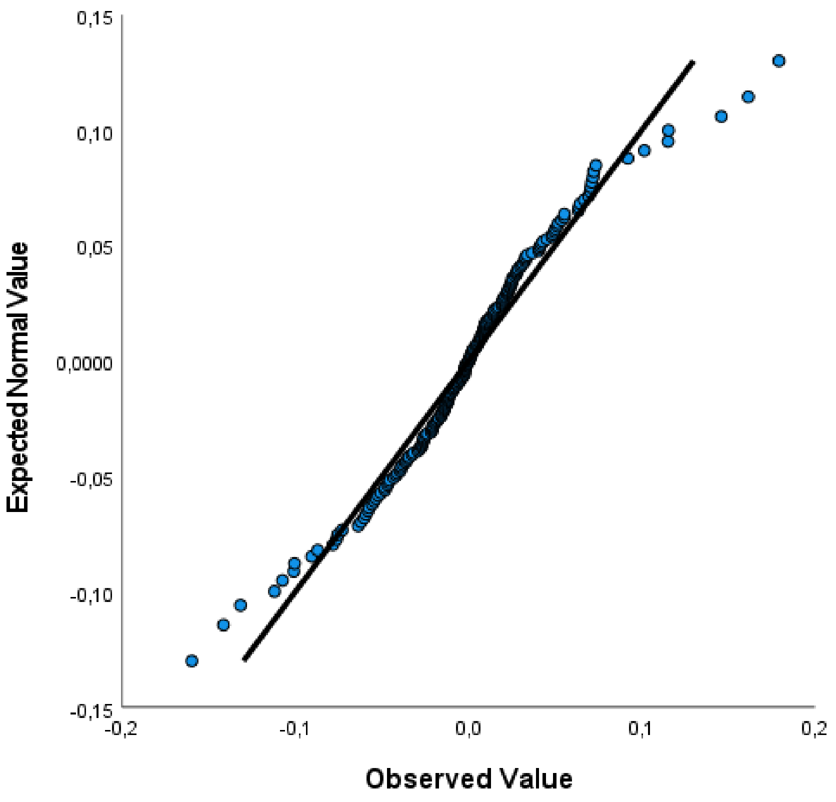

Figure S8 *LME Model Normal Q-Q Plot of Residuals.* This graph illustrates deviance of residuals of the linear mixed effects (LME) model of mean planning depth from normality (black line). Overall, the normality assumption is probably fulfilled.

Linear Regression Covariate Analysis

In a second analysis, we used backwards stepwise linear regression to identify predictors of subject-wise mean planning depths. For the initial model, we included performance in fluid cognitive abilities, i.e. performance in the IDP and the SWM, as well as SAT reaction time and a group indicator as predictors.

Model	R^2	Adj. R^2	*SE*	Change Statistics				
				R^2 Change	*F* Change	*df1*	*df2*	*p*
1	.410	.360	.2578	.410	8.179	4	47	<.001
2	.410	.373	.2552	<.001	.014	1	47	.908
3	.409	.385	.2528	-.001	.082	1	48	.776

Table S4 *Linear Regression Model Summary*

Model		Unstandardized Coefficients		Standardized Coefficients	*t*	*p*	95% CI for *b*	
		b	*SE*	*Beta*			*LL*	*UL*
1	Intercept	1.797	.285		6.309	<.001	1.224	2.370
	Group	-.253	.107	-.396	-2.367	.022	-.468	-.038
	SAT_RT (s)	.038	.011	.399	3.356	.002	.015	.061
	IDP_PER (%)	.000	.004	-.021	-.116	.908	-.009	.008
	SWM_PER (%)	.001	.002	.043	.306	.761	-.004	.006
2	Intercept	1.774	.206		8.594	<.001	1.359	2.190
	Group	-.245	.080	-.383	-3.047	.004	-.407	-.083
	SAT_RT (s)	.038	.011	.400	3.412	.001	.016	.061
	SWM_PER (%)	.001	.002	.036	.286	.776	-.004	.005
3	Intercept	1.828	.089		2.485	.000	1.648	2.007
	Group	-.254	.073	-.398	-3.497	.001	-.401	-.108
	SAT_RT (s)	.039	.011	.406	3.568	.001	.017	.060

Table S5 *Linear Regression Coefficients*. CI = confidence interval; *LL* = lower limit; *UL* = upper limit.

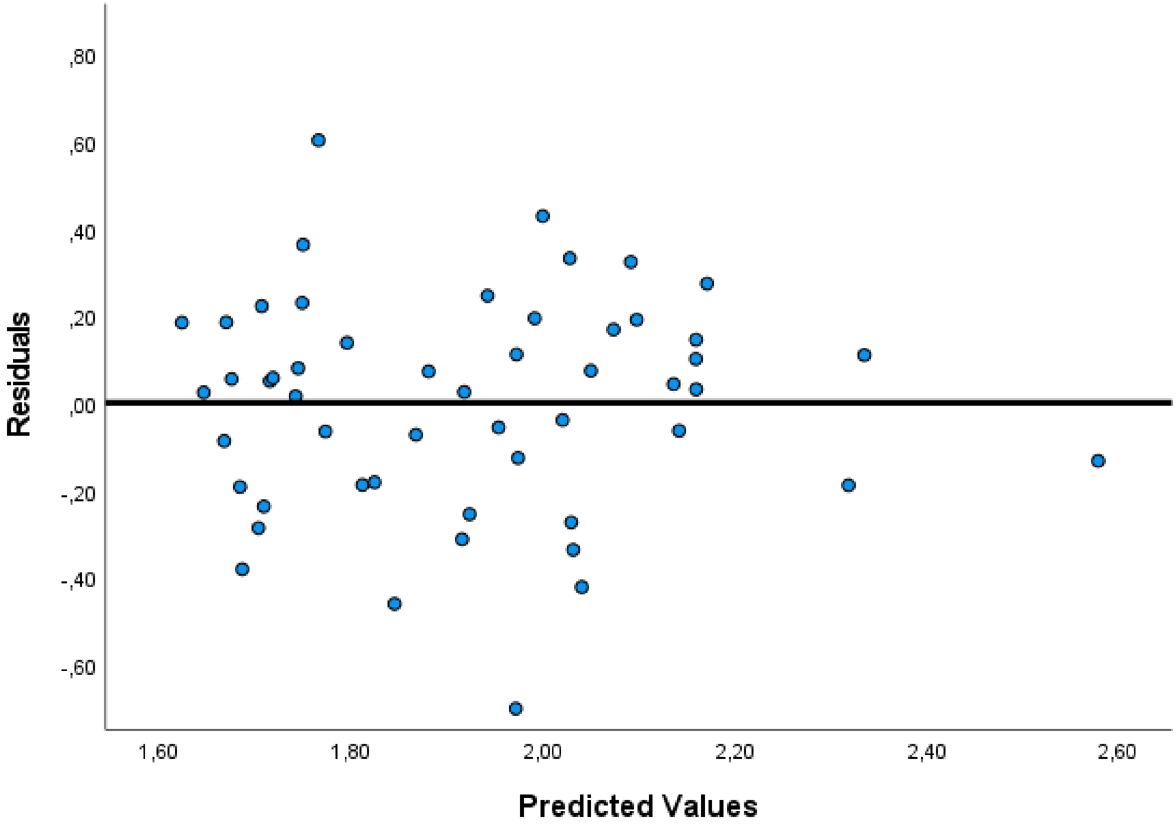

Figure S9 *Linear Regression Model 3 Residual Plot.* Scatterplot of residuals against predictive values of the linear regression model 3 of mean planning depth. There is no indication for a violation of the homoscedasticity assumption of residuals.

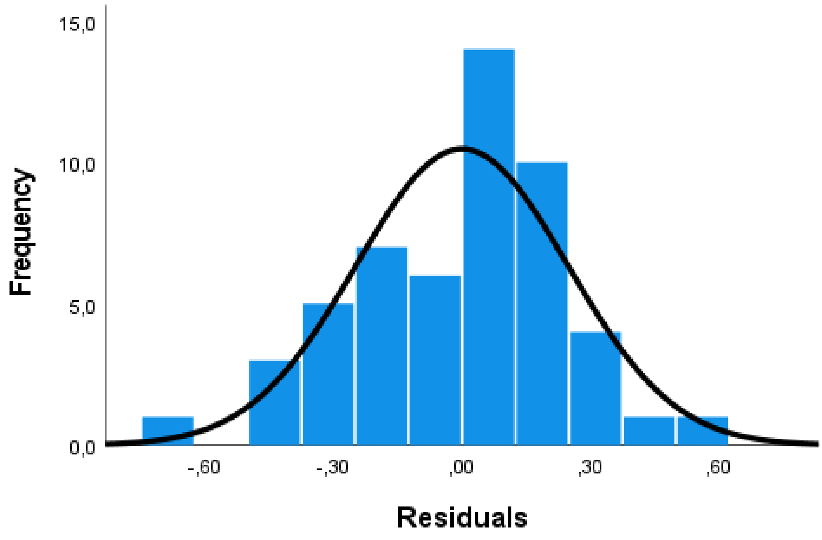

Figure S10 *Linear Regression Model 3 Histogram of Residuals.* The plots shows the histogram of residuals of the linear regression model of mean planning depth. The plot shows all residuals, i.e. for both groups and a fitted normal curve in black. Overall, residuals seem to be normally distributed.

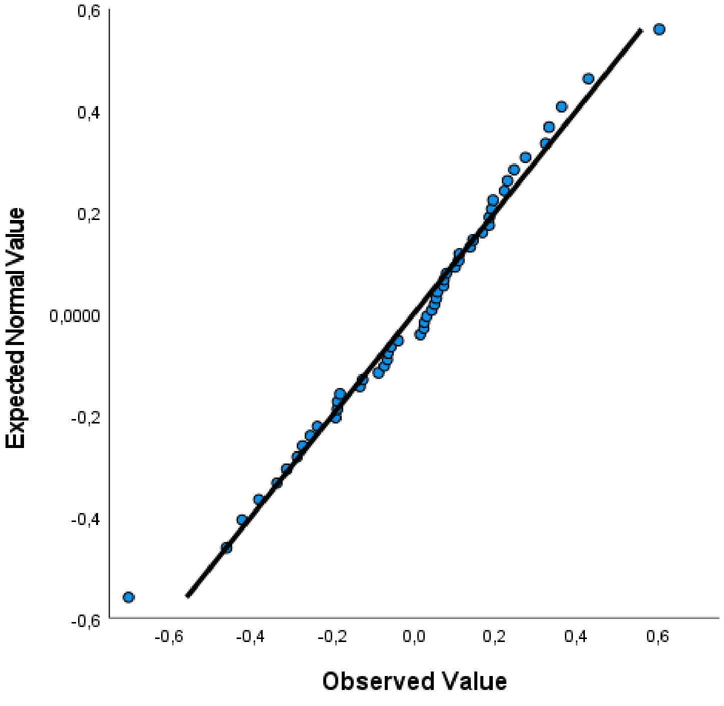

Figure S11 *Linear Regression Model 3 Normal P-P Plot of Residuals.* This graph illustrates deviance of residuals of the linear regression model of mean planning depth from normality (black line). It plots cumulative probabilities of model residuals (x-axis) against expected normal cumulative probabilities (y-axis). Overall, the normality assumption is probably fulfilled.

Linear Regression of SAT Performance

In a third analysis, we used linear regression to predict subject-wise task performance with the parameters of the computational Model.

Model	R^2	Adj. R^2	*SE*
1	.882	.872	6.877

Table S6 *Linear Regression Model Summary*

Model		Unstandardized Coefficients		Standardized Coefficients	*t*	*p*	95% CI for *b*	
		b	*SE*	*Beta*			*LL*	*UL*
1	Intercept	-31.777	7.287		-4.361	<.001	-46.438	-17.117
	alpha	-1.903	26.070	-.022	-.418	.678	-63.350	41.544
	beta	13.415	1.830	.500	7.331	<.001	9.734	17.097
	theta	-1.136	2.084	-.031	-.545	.588	-5.329	3.057
	MeanPD	31.475	4.582	.528	6.870	<.001	22.258	4.692

Table S7 *Linear Regression Coefficients.* CI = confidence interval; *LL* = lower limit; *UL* = upper limit; MeanPD = mean planning depth; alpha = learning rate, beta = inverse temperature; theta = response bias.

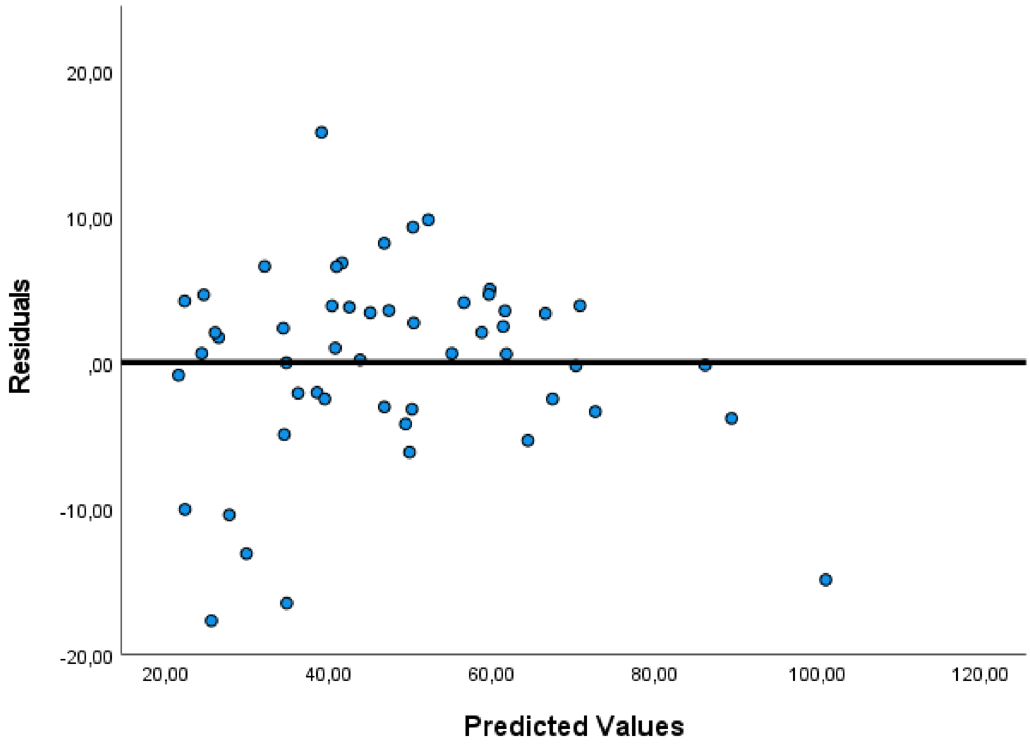

Figure S12 *Linear Regression Model Residual Plot.* Scatterplot of residuals against predictive values of the linear regression of performance in the Space Adventure Task. There is no indication for a violation of the homoscedasticity assumption of residuals.

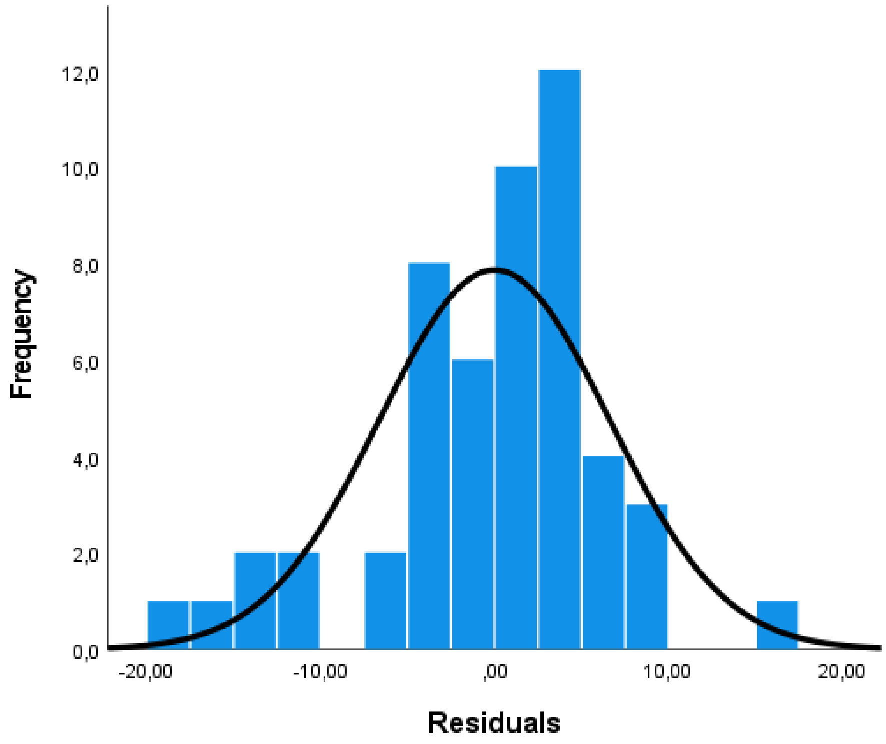

Figure S13 *Linear Regression Model Histogram of Residuals.* The plots shows the histogram of residuals of the linear regression of performance in the Space Adventure Task. The plot shows all residuals, i.e. for both groups and a fitted normal curve in black. Overall, residuals seem to be normally distributed.

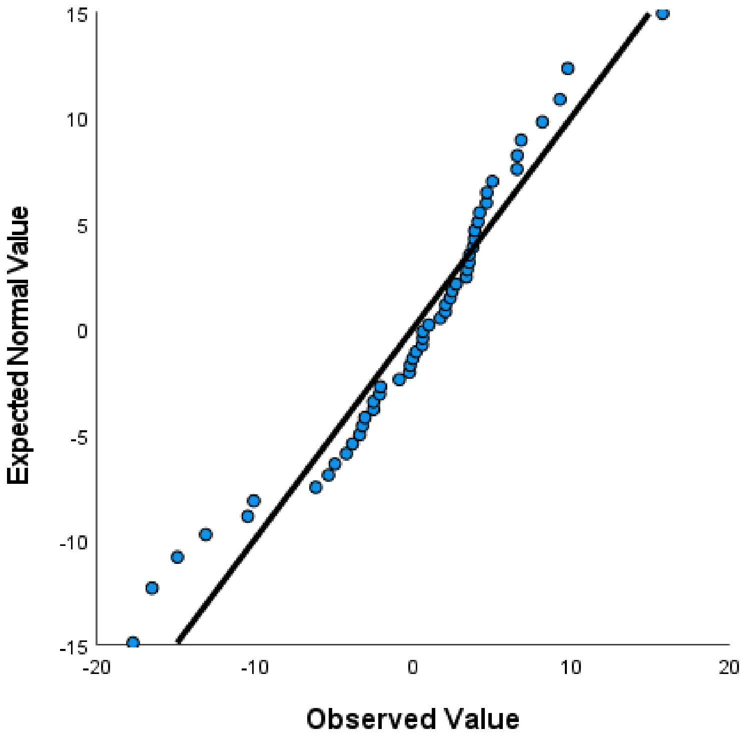

Figure S14 *Linear Regression Model Normal P-P Plot of Residuals.* This graph illustrates deviance of residuals of the linear regression model of performance in the Space Adventure Task from normality (black line). It plots cumulative probabilities of model residuals (x-axis) against expected normal cumulative probabilities (y-axis). Overall, the normality assumption is probably fulfilled.

References of Supplementary Material

1. Sutton, R. S. & Barto, A. G. *Reinforcement Learning: An Introduction*. (MIT press, 2018).
2. Jiang, N., Kulesza, A., Singh, S. & Lewis, R. The dependence of effective planning horizon on model accuracy. in *Proceedings of the 2015 International Conference on Autonomous Agents and Multiagent Systems* 1181–1189 (Citeseer, 2015).
3. Blei, D. M., Kucukelbir, A. & McAuliffe, J. D. Variational Inference: A Review for Statisticians. *J. Am. Stat. Assoc.* **112**, 859–877 (2017).
4. Bingham, E. *et al.* Pyro: Deep Universal Probabilistic Programming. *J. Mach. Learn. Res.* **20**, 973–978 (2019).
5. Satterthwaite, F. E. An Approximate Distribution of Estimates of Variance Components. *Biom. Bull.* **2**, 110–114 (1946).
